# Treatment patterns for AL amyloidosis after frontline daratumumab, bortezomib, cyclophosphamide, and dexamethasone treatment failures

**DOI:** 10.1038/s41375-024-02243-5

**Published:** 2024-04-09

**Authors:** Saurabh Zanwar, Morie A. Gertz, Eli Muchtar, Francis K. Buadi, Taxiarchis Kourelis, Wilson Gonsalves, Ronald S. Go, Suzanne Hayman, Prashant Kapoor, Moritz Binder, Joselle Cook, David Dingli, Nelson Leung, Yi Lin, Rahma Warsame, Amie Fonder, Miriam Hobbs, Yi Lisa Hwa, Robert A. Kyle, S. Vincent Rajkumar, Shaji Kumar, Angela Dispenzieri

**Affiliations:** Division of Hematology, Mayo Clinic, Rochester, MN US

**Keywords:** Haematological cancer, Myeloma

The immediate goal for therapy in patients with systemic immunoglobulin light chain amyloidosis (AL) is to swiftly achieve at least a hematologic very good partial response (VGPR), given the consistently improved organ responses and survival with achievement of deep hematologic responses [1–3]. The phase III ANDROMEDA trial demonstrated that the addition of daratumumab to bortezomib, cyclophosphamide, and dexamethasone (D-VCd) resulted in significantly higher hematologic CR (53%) and VGPR rates (78%) compared to VCd in previously untreated patients [4]. These outstanding response rates have led to D-VCd being the currently accepted frontline therapy for AL amyloidosis. However, up to one-fourth of patients do not achieve a deep response (≥VGPR) with D-VCd induction and may need subsequent treatment to improve the response depth [4]. Data guiding the selection of subsequent treatment in these patients are limited, and here we report on the patterns of treatment failure and subsequent therapies in this cohort of patients.

After the institutional review board approval, patients initiated on frontline D-VCd regimen between 01/2018 and 12/2022 were evaluated. Patients requiring a subsequent treatment due to suboptimal response [≤ partial response (PR)], progression, or toxicity were included in this study. Additionally, patients who received subsequent treatment for rising involved serum-free light chains (sFLC) after achieving a deep response (≥VGPR) without meeting the criteria for progression were also included. Preplanned autologous stem cell transplantation (ASCT) after achieving a deep response with D-VCd induction was not considered as second-line/subsequent therapy. The Mayo 2004 stage with the European modification (stage IIIB with NT proBNP > 8500 ng/L) was used for risk stratification, and laboratory values for troponin and BNP were harmonized using previously described conversions [5, 6]. Hematologic response was assessed from the start of subsequent treatment using the Consensus criteria [7]. For patients with dFLC between 2 and 5 mg/dL at initiation of subsequent therapy using, a low dFLC PR was categorized as a dFLC < 1 mg/dL [8]. Given the expected delay in achieving organ response, this endpoint was determined from the initiation of frontline treatment, with responses defined by the binary response criteria [1, 9]. Event-free survival (EFS) and overall survival (OS) were calculated from the start of subsequent treatment. For calculating the EFS, the events of interest included discontinuation of D-VCd treatment for progression or rising sFLC without meeting the progression criteria, suboptimal response, adverse effect, or death. All time-to-event analyses were conducted using the Kaplan–Meier method and compared using the log-rank test. Fischer’s exact test was used for comparison of nominal data and non-parametric tests were used to compare continuous data.

Of 119 patients treated with frontline D-VCd regimen during the study period, 28 (24%) patients required switching to a subsequent therapy. Patients requiring second-line therapy were younger (median age 62 years vs. 67.3 years, *p* = 0.042), had a higher proportion of patients with Mayo Stage I disease (41% vs 20%, *p* = 0.04), with a trend toward lower rates of Stage IIIB disease (4% vs 15%, *p* = 0.17) and higher rates of 1q gain/amplification (36% vs 18%, *p* = 0.06; Supplementary Table 1).

For the twenty-eight patients who received subsequent therapy, the median follow-up from the diagnosis of AL amyloidosis was 29 (95% CI: 25–36) months and the median follow-up from the start of subsequent therapy was 19.7 (95% CI: 16.4–26.7) months. Prior to the initiation of subsequent therapy, the median duration on D-VCd was 5.7 months (range 1.5–26 months). Six (21%) patients were receiving daratumumab maintenance at the time of initiation of subsequent therapy. Twenty-nine percent (*n* = 8) patients had Mayo Stage III disease and 68% (*n* = 19) patients had >10% bone marrow plasma cell infiltrate at diagnosis. The patient characteristics and best hematologic response to D-VCd are depicted in Table 1. The most common reason for the change in treatment was suboptimal response to D-VCd in 22 (79%) patients [PR in 20 patients, no response (NR) in 2 patients], hematological progression in 3 (11%) patients, rising sFLC from VGPR in 2 (7%) patients and treatment-emergent adverse effect (peripheral neuropathy) in 1 (4%) patient.

**Table 1:** Patient characteristics and treatment details for the cohort of patients receiving a subsequent treatment after D-VCd (*n* = 28).

Median Age at diagnosis of AL Amyloidosis, years (range)	62 (23–82)
AL subtype, *n* (%)	
IgG	12 (43)
IgA	5 (18)
Light chain only	11 (39)
Light chain subtype, % Lambda	68
Mayo 2004 staging with European modification, *n* = 27 (%)	
Stage I	11 (41)
Stage II	8 (27)
Stage IIIA	7 (24)
Stage IIIB	1 (4)
Chromosomal abnormalities at diagnosis, *n* (%)	
*t*(11;14)	12 (43)
1q gain/amplification	10 (36)
Hyperdiploid	6 (21)
Deletion 13q	5 (18)
High-risk chromosomal abnormalities [deletion 17p, *t*(4;14), *t*(14;20)]	3 (11)
Organ involvement, *n* (%)	
Cardiac	13 (46)
Renal	15 (54)
Gastrointestinal	6 (21)
Liver	3 (11)
Peripheral neuropathy	3 (11)
Hematologic response at initiation of first salvage therapy after D-VCd, *n* (%)	
Complete response	0
Very good partial response^a^	2 (7)
Partial response	20 (68)
No response	3 (11)
Progression	3 (11)
Second-line treatment regimens, *n* (%)	
Daratumumab (D)-based combination	11 (39)
DPd	4 (14)
DRd	2 (7)
D + PI+IMiD (D-KPd, D-VPd)	2 (7)
D + PI+Alkylator (D-KCd, D-VMP)	2 (7)
D-Bendamustine	1 (4)
Autologous stem cell transplantation	8 (29)
BCL2 inhibitor- based	6 (21)
Ven-dexamethasone (dex)	3 (11)
Ven-bortezomib-dex	1 (4)
Ven-pomalidomide-dex	1 (4)
Lisaftoclax(APG-2575)-pomalidomide-dex	1 (4)
Others (PCyD, PVd, K-rituximab-methylprednisone)	3 (11)

*D* daratumumab, *D-VCd* daratumumab, bortezomib, cyclophosphamide, dexamethasone, *IMiD* immunomodulatory drugs, *PI* proteasome inhibitors, *DPd* daratumumab, pomalidomide, dexamethasone, *DRd* daratumumab, lenalidomide, dexamethasone, *K* carfilzomib, *KCd* carfilzomib, cyclophosphamide, dexamethasone, *VMP* bortezomib, melphalan, prednisone, *Ven* venetoclax, *PCyD* pomalidomide, cyclophosphamide, dexamethasone, *PVd* pomalidomide, bortezomib, dexamethasone, *CR* complete response, *VGPR* very good partial response, *PR* partial response, *NR* no response, *PD* progressive disease.

^a^Two patients achieved a VGPR but subsequently demonstrated rising serum-free light chains (not meeting criteria for progression), hence switched to subsequent treatment.

**Fig. 1:**
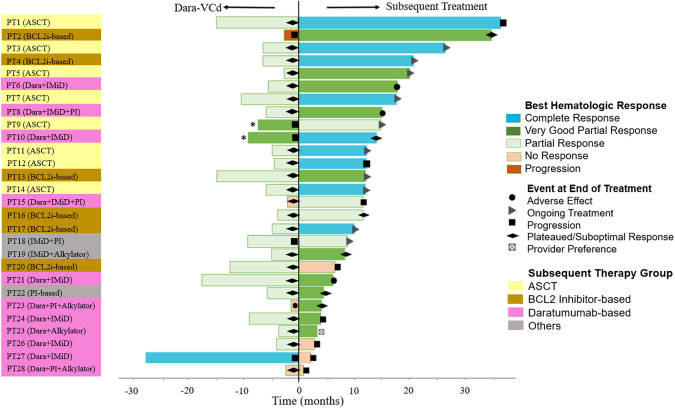
Swimmer plot depicting details of subsequent treatment, best hematologic response to preceding Daratumumab, bortezomib, cyclophosphamide, and dexamethasone (D-VCd), reason for switch to subsequent therapy and status at last follow-up for subsequent therapy. ASCT autologous stem cell transplant, BCL2i BCL2 inhibitor, Dara daratumumab, IMiD immunomodulatory drugs, PI proteasome inhibitor, PT patient, Ven venetoclax. *These patients demonstrated rising serum-free light chains without meeting the formal progression criteria.

Subsequent therapies were grouped into 4 cohorts: daratumumab-based combinations (*n* = 11, 39%), ASCT (*n* = 8, 29%), BCL2 inhibitor (BCL2i)-based therapies (*n* = 6, 21%), and miscellaneous therapies, not classified (*n* = 3). The details of the salvage therapies are included in Table 1. Figure 1 shows a swimmer plot with the treatment responses. Among patients switching from D-VCd due to suboptimal hematologic response (*n* = 22), 19 (86%) patients achieved a deeper magnitude of hematologic response with subsequent therapy. The best hematologic response with subsequent therapy was CR in 9 (32%) patients, VGPR in 12 (43%) patients, PR in 3 (11%) patients (1 patient achieved a low dFLC PR), and no response was noted in 4 (14%) patients. The rate of hematologic CR was 63% with ASCT, 50% with BCL2i-based regimens, 10% with daratumumab-based regimens, and 0% for the 3 patients receiving other therapies (*p* = 0.057). Of 24 organ-response evaluable patients, 11 (46%) demonstrated response in at least 1 organ. Five of the thirteen patients with cardiac involvement (38%) had a cardiac response, and 7 of the fifteen patients (47%) patients with renal involvement achieved a renal response.

At last follow-up, 10 (36%) patients had ongoing response to subsequent treatment, 8 (29%) patients progressed, 6 (21%) patients stopped the second-line treatment due to plateauing of response after an initial hematologic response, 3 (11%) patients stopped treatment due to adverse events, and 1 (4%) patient switched treatment due to physician preference. The median EFS with the second-line treatment was 14.3 months [95% CI: 11.4 months-not reached]. The median EFS was 36.3 months (95% CI: not reached-not reached) for the ASCT cohort, 34.9 months (95% CI: 11.4-not reached) for BCL2 inhibitor-based regimen cohort, 6.2 months (95% CI: 3.8-not reached) for daratumumab-based combinations, and 8.3 months (95% CI: 4.3-not reached) for the miscellaneous group (*p* = 0.001, Supplementary Fig. 1). The effect of treatment group on EFS was independent of the Mayo Stage at diagnosis. Patients receiving daratumumab-based salvage regimens had worse EFS [HR 3.7 (95% CI: 1.2–11.4), *p* = 0.02] compared to non-daratumumab-based regimens. At the time of last follow-up, 3 patients died and the 18-month OS rate was 89%.

The ANDROMEDA study and recent retrospective series report rapid and deep responses with daratumumab-based treatments, with reduction in early mortality rates [10]. However, as demonstrated in our cohort and the ANDROMEDA study, 20–25% of the patients do not achieve a deep hematologic response with D-VCd and required a subsequent therapy for treatment failure, most commonly due to suboptimal response [4]. The treatment options for AL amyloidosis have expanded significantly in the past decade, with the repurposing of various treatments for multiple myeloma in patients with AL amyloidosis [11]. Case series demonstrate that venetoclax leads to deep hematologic and organ responses in patients with relapsed/refractory AL amyloidosis patients harboring *t*(11;14) [12]. There are exciting data on the role of B cell maturation antigen targeting bispecific antibody, teclistamab, in AL amyloidosis [13]. These results suggest potentially viable options for managing D-VCd failures.

The dearth of data regarding optimal treatment post-D-VCd failures, be it inadequate hematologic response or overt progression, prompted the present study. It was reassuring to find that D-VCd failures respond to subsequent therapies. In most patients in our cohort, the hematologic response deepened with the use of subsequent therapy, suggesting the value of switching therapy in case of suboptimal hematologic response. It is well established that achieving deep hematologic response is a predictor for organ response and OS, and therefore an important initial goal of treatment [14]. The cardiac and renal response rates in our series were comparable to the 6-month organ response rates reported in the ANDROMEDA study, but longer follow-up is needed for a detailed assessment [4].

Our data suggest that continuing daratumumab with the addition of IMiDs (pomalidomide, lenalidomide) and/or the second-generation proteasome inhibitor, carfilzomib is not a suitable option, although results could be confounded by cohort heterogeneity with a small sample size and possible selection bias. The better options for consolidation for patients with suboptimal hematologic response appeared to be either BCL2 inhibitor (predominantly venetoclax)-based therapy for *t*(11;14) AL or ASCT, with the caveat that only select patients will be candidates for either of these options. As one of several examples of improved outcomes with additional therapy after suboptimal response, consolidation after ASCT for those achieving less than a VGPR has resulted in higher rates of deep hematologic response and improved OS [15].

Within the limitations of the retrospective nature of the study and variable individual practice patterns, our findings demonstrate a clear superiority in both the CR rates and the EFS with ASCT and BCL2 inhibitor-based treatments compared daratumumab-based combinations. The restriction of the utility of venetoclax in patients harboring t(11;14) and the fitness of patients for the ASCT-based approach limit the applicability of our findings to specific patient populations. Nonetheless, when feasible, non-daratumumab-based regimens likely represent the preferred subsequent treatment option for patients with treatment failure to frontline D-VCd. Given the fact that most patients who will achieve VGPR with D-VCd achieve it within 2 months, alternative therapies could be considered at this time point in the setting of suboptimal hematologic response.

### Supplementary information

Supplementary Material
