# Supplementary material for: Treatment patterns for AL amyloidosis after frontline daratumumab, bortezomib, cyclophosphamide, and dexamethasone treatment failures
Source: Leukemia. 2024 Apr 9;38(6):1423–6. doi: 10.1038/s41375-024-02243-5 (PMC11147750; doi:10.1038/s41375-024-02243-5)
Supplement: Supplementary file 1 — Supplementary Material [file 41375_2024_2243_MOESM1_ESM.docx]

| Supplementary Table 1. Comparison of baseline characteristics of patients receiving a second-line treatment versus not after D-VCd | | | |
| --- | --- | --- | --- |
| Clinical Parameter | Requiring second line therapy  (n=28) | Not requiring subsequent therapy  (n=91) | p-value |
| Age, median, years (range) | 62 (23-82) | 67.3 (41-83) | **0.042** |
| Light Chain Isotype, % lambda | 68 | 83 | 0.1 |
| ECOG Performance Status >1, n (%) | 5 (20) | 21 (25) | 0.8 |
| NYHA class >2, n (%) | 4 (17) | 18 (23) | 0.6 |
| Mayo 2004 Stage with European Modification | | | |
| I | 11 (41) | 16 (20) | **0.04** |
| II | 8 (27) | 25 (32) | 1.0 |
| IIIA | 7 (25) | 26 (33) | 0.63 |
| IIIB | 1 (4) | 12 (15) | 0.17 |
| Organ Involvement | | | |
| Cardiac | 13(46) | 61 (67) | 0.07 |
| Renal | 15 (54) | 59 (65) | 0.37 |
| Gastrointestinal | 6 (21) | 18 (20) | 0.79 |
| Liver | 3 (11) | 4 (4) | 0.35 |
| Neuropathy | 3 (11) | 21 (23) | 0.18 |
| Number of organs involved, median | 1 | 2 | 0.14 |
| Chromosomal Abnormality | | | |
| t(11;14) | 12 (43) | 34 (46) | 0.82 |
| 1q gain/amplification | 10 (36) | 13 (18) | **0.06** |
| Hyperdiploid | 6 (21) | 4 (5) | 0.21 |
| Deletion 13q | 5 (18) | 22 (31) | 0.22 |
| High-risk [deletion 17p, t(4;14), t(14;20)] | 3 (11) | 8 (11) | 1.0 |
| Bone marrow plasma cell infiltrate >10%, % | 68 | 43 | **0.02** |
| 30-month OS from start of D-VCd, % | 89 | 83 | 0.45 |
| D-VCd: daratumumab, bortezomib, cyclophosphamide, dexamethasone; ECOG: Eastern Cooperative Oncology Group; NYHA: New York Heart Association; OS: overall survival | | | |


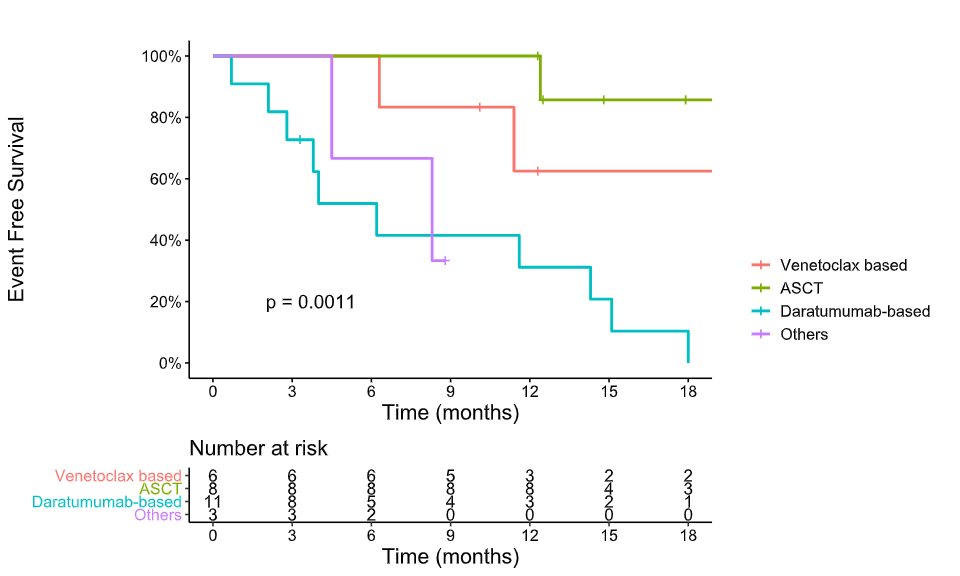


**Supplementary Figure 1.** Event-free survival for subsequent treatment after D-VCd demonstrates significantly better EFS for patients treated with ASCT or venetoclax-based regimens, with daratumumab-based regimens demonstrating inferior EFS.
